# Perceptions of research experience during internal medicine training: insights from a national survey

**DOI:** 10.1080/07853890.2025.2534848

**Published:** 2025-07-24

**Authors:** Laith Alhuneafat, Randa Mahasneh, Nada Alrifai, Jillian S. Catalanotti, Gopal Yadavalli, Anastasios Kapetanos

**Affiliations:** ^a^ Cardiovascular Disease Institute, University of Minnesota, Minneapolis, MN, USA; ^b^ Department of Educational Psychology, Faculty of Educational Sciences, The Hashemite University, Zarqa, Jordan; ^c^ Department of Rheumatology, Cooper University, Camden, NJ, USA; ^d^ Department of Medicine, The George Washington University School of Medicine and Health Sciences, Washington, DC, USA; ^e^ Department of Medicine, Boston University Chobanian and Avedisian School of Medicine, Boston, MA, USA; ^f^ Department of Medicine, Allegheny Health Network, Pittsburgh, PA, USA

**Keywords:** Publication pressure, internal medicine, graduate medical education, scholarly activity

## Abstract

**Background:**

There is limited insights into how internal medicine (IM) residents perceive research. This study aimed to assess residents’ perceptions across five domains: well-being, research competence, research support, effect on training, and research quality.

**Methods:**

In 2022, a survey instrument was developed to assess residents’ perceptions of research across five domains. All IM programs listed in Fellowship and Residency Electronic Interactive Database (FREIDA) were contacted by email, and residents were invited to participate.

**Results:**

A total of 530 residents from 67 IM programs completed our survey. Just over 80% of respondents had participated in research during residency. Residents were dissatisfied with research opportunities and infrastructure but viewed research funding more positively. Negative views were held regarding the impact of publication pressure on well-being and the quality of research produced. Publication pressure was perceived to compromise clinical work and limit career paths. Some residents acknowledged that research enriched their training. Residents who had already matched into a fellowship program reported higher research competency and better impressions of the impact of research on their medical training compared to those who had not yet or did not plan to match into a fellowship. Residents who planned to prioritize research in their future careers held positive perceptions of research across multiple domains.

**Conclusions:**

This study provides valuable insights into the impact of publication pressure on IM residents. The findings suggest a need to realign expectations for research productivity or target interventions to better support research for all residents, regardless of track or career goals.

## Background

Participating in scholarly activity during residency is an Accreditation Council for Graduate Medical Education (ACGME) requirement, fulfilled by activities as varied as presenting at journal clubs or serving as a journal editor [1]. Participation in research enhances career prospects, including the likelihood of securing preferred fellowship positions [2–5]. Considering the competition for internal medicine (IM) fellowships, residents may perceive research as a means to differentiate themselves from other applicants [5–7].

Teaching scientific thinking is most effective when medical trainees are involved in scientific research [8]. Anaya-Prado et al. argue that the experience of conducting research and publishing the results is a vital prerequisite to physicians effectively evaluating other scientific studies [9]. Publishing research results during medical school and residency has been shown to correlate with continued publication throughout the physician’s career [10]. A strong publication record, in turn, can contribute to career advancement. The skills developed through research and writing may provide a counterbalance to the detrimental aspects of pressure to publish (‘publication pressure’). However, the demands of pursuing research, combined with other residency responsibilities, may detract from the quality of a trainee’s clinical training, patient care, or well-being, and contribute to burnout [1,3,4]. Additionally, publication pressure is a known driver for students to engage in academic misrepresentation and erroneous claims of publications [11,12].

Understanding the pressures residents face in conducting and publishing research is crucial for reshaping residency programs so that their scholarly expectations align with actual training demands. By examining residents’ reported challenges, institutions can adapt support structures and redefine publication goals. This study investigates publication pressure through residents’ perceptions of research and publication with regards to well-being, research competence, research support, effect on medical training, and quality of research produced. Additionally, we explore potential factors that influence residents’ perceptions, including resident demographics, program details, and other motivating factors, as well as if publication pressure influences career choices.

## Methods

### Sample and participants

Potential participants included residents at all IM and combined IM residency programs with a program email address listed on the American Medical Association’s Fellowship and Residency Electronic Interactive Database (FREIDA) website [13].

### Survey development

We adapted validated survey tools for our study and conducted pilot testing with a small group of IM residents for reliability and validity [14,15]. Those in the pilot were excluded from the final sample. Feedback refined survey questions and improved content validity. Participation in research was defined for respondents as any active engagement in scholarly work during residency (completed or ongoing). The survey assessed residents’ perceptions across domains of well-being, research competence, support, impact on training, and research quality. It was comprised of 27 five-point Likert scale statements categorized into five domains and administered *via* Microsoft Forms. The mean scores were computed within each domain. Low scores indicate low agreement with the statement while higher scores indicated strong agreement with the statement. The survey details can be found in the supplemental file.

### Data collection

We emailed all 451 IM and combined IM residency programs and requested that they forward our electronic invitation to participate, including a link to the survey, to all current residents. After two weeks, a reminder email was sent to all programs. The survey was anonymous, with no collection of identifying information, and participation was voluntary. To submit the survey, participants were required to answer all of the questions. The survey was available for participation between May 23 and June 23, 2022.

### Data analysis

We excluded responses with negligible variances where participants consistently chose the same response, irrespective of content or direction. Scores for negatively worded statements were reversed, and internal consistency was assessed using Cronbach’s alpha. Percentages, mean, and standard deviation (SD) were calculated for each survey domain. One-sample t-tests (*p* < 0.01) determined if mean scores significantly differed from the neutral value of 3.

We conducted a factorial analysis of variance (ANOVA) on the scores from all respondents for each domain of the survey. We explored whether perceptions differed based on several variables, including training level, race/ethnicity, gender, residency program type, fellowship match status, visa requirement, type of medical school attended, and intention to prioritize research in the future. Post-hoc comparisons were carried out using the least significant difference method. Significance was set at *p* < 0.006 after Bonferroni correction for multiple comparisons. All analyses were performed using IBM SPSS V27 Statistics.

### Ethical considerations

The study received ethical approval from the Allegheny Health Network Research Institutional Review Board before data collection(2022-079-AGH). All participants provided informed written consent before participating in the study. The study ensured the confidentiality and anonymity of participants by not collecting any personal information. The study adhered to the ethical principles outlined in the Declaration of Helsinki [16].

## Results

### Internal consistency of the instrument

Supplemental Table S1 displays the Cronbach’s alpha values (range from 0.64 ‘research quality’ to 0.82 ‘effect on well-being’). These values indicate acceptable to good reliability. Corrected item-total correlations were above 0.30, suggesting that each item within its respective domain consistently measures the intended construct [17].

### Participants

Sixty-seven of 451 programs shared our survey, yielding an 11.8% response rate with 530 trainee respondents out of 4,492 potential participants. Institutes that did not share our survey were not included as potential respondents. We excluded 48 non-categorical IM or IM combined trainee responses and dropped 50 responses with negligible variances. The final sample size was 432 respondents from programs from multiple states. Most represented were Pennsylvania (15.5%), Massachusetts (11.3%), New York (10.4%), Ohio (9.3%), Connecticut (7.4%), Texas (6.9%), and California (4.9%).

Participants were evenly split by gender, were mostly Asian (37.3%) or White (35.6%), and the majority were aged 26-30 (69.7%). Respondents spanned all levels of training: post-graduate-year (PGY)-1 = 144 (33.3%), PGY-2 = 156 (36.1%), PGY-3 = 127 (29.4%), and PGY-4 or higher = 5 (1.2%). Approximately half trained at university-based residency programs (48.8%), and the majority did not require a visa (70.8%) (Table 1). Career interests varied; common choices included hospital medicine (16.52%), cardiology (13.73%), hematology-oncology (9.66%), gastroenterology (9.01%), pulmonology and critical care (8.37%), and primary care (7.94%) (Supplemental Table S2).

**Table 1.** Demographics and professional experiences of study participants (*n* = 432).

Variable	Levels	n (%)
Gender	Male	211 (48.8)
	Female	216 (50.0)
	Non-Binary	2 (0.5)
	Prefer not to say	3 (0.7)
Race	Asian	161 (37.3)
	Black or African American	23 (5.3)
	Hispanic or Latino	42 (9.7)
	White	154 (35.6)
	Other	21 (4.9)
	Prefer not to say	31 (7.2)
Training Level	PGY1	144 (33.3)
	PGY2	156 (36.1)
	PGY3	127 (29.4)
	PGY4 or greater	5 (1.2)
Residency Program	Community Based	116 (26.9)
	Community Based, University Affiliated	105 (24.3)
	University Based	211 (48.8)
Location of residency program (State abbreviation)	Northeast (PA, NY, NJ, CT, MA, RI, VT, NH, ME)	211 (48.8)
	Midwest (ND, SD, NE, KS, MO, IA, MN, IL, WI, IN, MI, OH)	88 (20.4)
	South (TX, OK, AR, LA, MS, AL, TN, KY, GA, FL, SC, NC, WV, VA, DC, MD, DE)	91 (21.1)
	West (WA, OR, ID, MT, WY, CO, UT, NV, CA, AZ, NM, AK, HI)	42 (9.7)
Matched into Fellowship	No	376 (87)
	Yes	56 (13)
Age group	<25 yrs.	4 (0.9)
	26-30 yrs.	301 (69.7)
	31-35 yrs.	109 (25.2)
	36-40 yrs.	11 (2.5)
	41-45 yrs.	3 (0.7)
	46 yrs. and greater	1 (0.2)
	Prefer not to say	3 (0.7)
Required to apply for a visa?	No	306 (70.8)
	Yes	120 (27.8)
	Prefer not to say	6 (1.4)
Residency type	Categorical Internal Medicine Resident	407 (94.2)
	Med-Peds Resident	18 (4.2)
	Other Med-Combined programs	7 (1.6)
Medical school	Allopathic US Medical School (MD)	159 (36.8)
	Caribbean Medical School (MD)	45 (10.4)
	International Medical School (MD/MBBS)	145 (33.6)
	Osteopathic US Medical School (DO)	81 (18.8)
	Other	2 (0.5)
Participation in research during residency	No	83 (19.2)
	Yes	349 (80.8)
I plan to make research an important part of my future career	Strongly Agree	44 (10.2)
	Agree	88 (20.4)
	Neither Agree nor Disagree	110 (25.5)
	Disagree	140 (32.4)
	Strongly Disagree	50 (11.6)
No. of projects published during residency	0	89 (20.6)
	1-5	219 (50.7)
	6-10	26 (6.0)
	11-15	7 (1.6)
	>15	8 (1.9)
	Prefer not to say	83 (19.2)

PGY: Postgraduate year.

### Participation in research

The majority of respondents (80.8%) participated in research during residency, even if it had not yet been published or presented. The majority had published 1-5 projects (50.7%). Research participation differed significantly by PGY level (*p* < 0.001), with higher engagement among PGY2 and PGY3 residents. Residents who matched into fellowship were also significantly more likely to have participated in research (*p* < 0.001) (Supplemental Table S3). About one-third of participants did not plan for research to be an important part of their future career (32.4%).

### Perception of research experiences during training

The mean value of scores in the domains of research support, research competence, and effect on medical training did not significantly differ from the neutral value of 3 (Supplement Table S4). Respondents reported a significant negative impact of publication pressure on well-being (*p* < 0.001) and perceived research quality (*p* < 0.001). The highest agreement was observed in satisfaction with research support (mean = 3.08, SD = 0.75). The lowest agreement was observed in achievement of research productivity without compromising quality (mean = 2.42, SD = 0.64).

**Figure 1.**
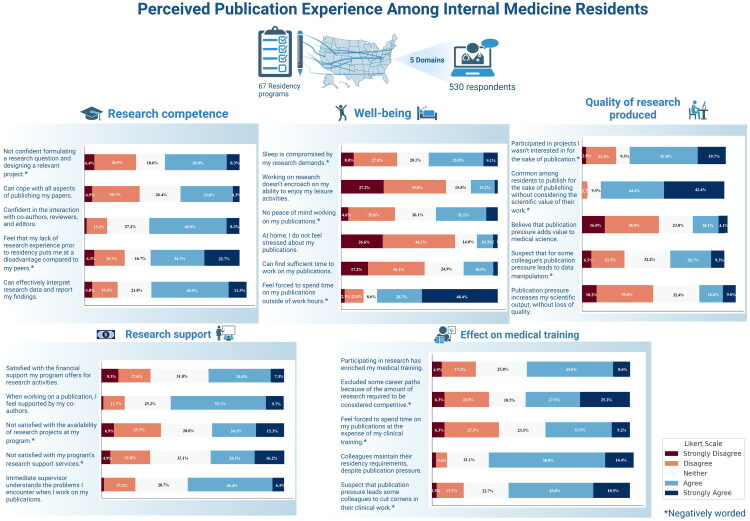
The perception of research experience by internal medicine residents.

Co-authors were the primary source of research support with 62% of participants expressing agreement or strong agreement, with supervisors following at 53% (Supplemental Table S1 and Figure 1). Satisfaction with financial support was 41%, while satisfaction with research project availability and program research support services stood at 33% and 27%, respectively.

Residents’ perceptions of their research competency varied. Fifty-seven percent felt disadvantaged due to limited pre-residency research experience. Although 61% were confident in interpreting data, only 38% believed they could handle all aspects of publishing.

Regarding the impact of research on medical training, 61% of respondents believed that publication pressure contributed to cutting corners in clinical work, and 53% believed they had to exclude some career paths due to expectations for research. Forty-three percent believed they sacrificed clinical training to achieve publications. Approximately 53% believed research enriched their training, and 73% thought their colleagues met general residency requirements despite publication pressure.

Regarding well-being, 77% worked on publications outside work hours and caused stress at home for 73%. Leisure activities were affected for 67%. Fifty-three percent struggled finding time for writing publications, 44% experienced compromised sleep, and 40% lacked peace of mind while working on publications.

Participants reported negative effects of publication pressure on research quality: 87% published without considering scientific value, 68% engaged in projects that did not interest them for the sake of publishing, 54% believed it did not add value to medical science, and 50% thought it increased scientific output at the expense of quality. Additionally, 38% suspected colleagues manipulated data due to publication pressure.

### Influence of participant factors on research during residency

Respondents’ perceptions of research during medical training differed significantly by race, fellowship match status, and future career plans in research. Those who had already matched into a fellowship program reported higher levels of research competence compared to those who had not yet matched or did not plan to apply (Mean = 3.43, SD = 0.70 versus Mean = 2.99, SD = 0.76; *F* = 10.771, *p* < 0.006). Respondents that had matched into a fellowship also perceived more positive effects of research on their medical training compared to residents who had not yet or did not plan to match (Mean = 3.24, SD = 0.65 versus Mean = 2.96, SD = 0.68; *F* = 7.772, *p* < 0.006).

Significant differences were observed in self-perception of research competence based on race (*F* = 3.76, *p* < 0.006) (Table 2). To further investigate these differences, post-hoc comparisons using the least significant difference were conducted. Black/African American respondents reported higher levels of research competence compared to Asian residents (Mean = 3.40, SD = 0.78 versus Mean = 2.95, SD = 0.77). However, perceptions of research competence among residents from other racial backgrounds were similar (Supplemental Table S5).

**Table 2.** Factorial ANOVA to test the difference in the perceptions of internal medicine residents regarding each domain of their experience in research according to demographics and career plans.

Domain	Variable	SS	F
Research Competence	Training Level	2.435	2.635
	Race	6.941	3.756*
	Gender	3.176	6.873
	Residency Program	1.093	1.183
	Fellowship	4.976	10.771*
	Visa	0.001	0.002
	Medical School	3.913	2.823
	Future Career	28.721	15.541*
Research Support	Training Level	1.559	1.488
	Race	5.006	2.389
	Gender	0.466	0.890
	Residency Program	2.667	2.546
	Fellowship	2.160	4.124
	Visa	1.053	2.010
	Medical School	0.849	0.541
	Future Career	0.814	0.388
Effect on Well-being	Training Level	1.343	1.169
	Race	2.316	1.008
	Gender	0.040	0.070
	Residency Program	4.064	3.537
	Fellowship	1.066	1.855
	Visa	0.528	0.918
	Medical School	0.078	0.045
	Future Career	8.708	3.789*
Research Quality	Training Level	0.037	0.051
	Race	1.898	1.330
	Gender	1.692	4.743
	Residency Program	1.914	2.683
	Fellowship	0.079	0.223
	Visa	0.017	0.047
	Medical School	1.180	1.102
	Future Career	13.151	9.217*
Effect on Medical Training	Training Level	1.092	1.458
	Race	4.121	2.752
	Gender	0.996	2.660
	Residency Program	0.080	0.106
	Fellowship	2.909	7.772*
	Visa	0.428	1.144
	Medical School	0.193	0.172
	Future Career	29.247	19.533*

SS (Sum of Squares): Measures the total variation attributable to each factor in the analysis.

F (F-ratio): The test statistic used in ANOVA, computed by dividing the MS of a factor by the MS of the error term; higher values suggest a greater likelihood that the observed effect is statistically significant.

* *p* ≤ 0.006.

Significant differences existed in respondents’ perceptions based on their intentions to prioritize research in their future careers. These differences were found in research competency (*F* = 15.54, *p* < 0.006), well-being (*F* = 3.79, *p* < 0.006), research quality (*F* = 9.22, *p* < 0.006), and impact on medical training (*F* = 19.53, *p* < 0.006) (Table 2). Post-hoc analysis revealed that residents intending to pursue research careers had more positive perceptions in these areas compared to other residents (Supplemental Table S6).

## Discussion

This study represents the first comprehensive investigation of IM residents’ research experiences and perceptions by exploring their impact on five key domains: well-being, research competence, research support, effect on medical training, and quality of research produced. The study sample was diverse in terms of gender, race, age, training level, residency program type, and future career plans of respondents.

Our analysis revealed several significant themes in how respondents perceive the pressure to publish (Figure 1). First, respondents expressed dissatisfaction with availability of research projects, and research support services available to them while having more favourable views about financial support. Second, they held negative views regarding the impact of research on their well-being and the quality of research produced. Third, publication pressure was perceived to compromise clinical work and limit career paths, although some residents acknowledged that research enriched their training while still allowing them to fulfil broad residency training expectations. Fourth, respondents who had already matched into a fellowship program reported higher research competence and perceived more positive effects of research on their medical training compared to those who had not yet matched or did not plan to apply. Lastly, respondents who intended to prioritize research in their future careers reported more positive perceptions of research across various domains. These findings underscore the need to balance research demands and clinical responsibilities in residency, promoting a culture that values high-quality research and resident well-being.

Programs should provide sufficient support and resources to help residents manage research workload and address associated stressors effectively. The US faces a shortage of physician-scientists [18,19] and participating in research during training can foster a desire to pursue a career as a research investigator [20–22]. Additionally, research cultivates critical thinking, sparks intellectual curiosity, and nurtures lifelong learning skills, all of which are indispensable in this era of evidence-based medicine [23–25]. Our findings and other recent studies demonstrate higher rates of research participation during training compared to earlier reports [26–28]. The increased participation of residents in research may be due to intrinsic motivation, external pressures such as career goals and fellowship aspirations, and improved institutional support [5,29].

### Research support and competency

Residents’ research activity is influenced by several factors. A significant limitation for resident investigators is scarce research mentorship, often attributed to a lack of trained faculty [30]. Additionally, residents express concerns about faculty lacking time and specific curricula for research mentorship [31]. In our study, 62% of participants identified co-authors as the primary sources of support during their research endeavours. A lower, but still encouraging, 53% felt their immediate supervisors fully understood the challenges they faced while working on publications. A key element for a thriving research program is the presence of a substantial and effective mentoring pool [32]. This includes diverse types of mentors, which not only alleviates mentor fatigue, but also offers greater flexibility for both residents and mentors [32]. The low satisfaction among our participants regarding the availability of research projects likely relates to the available mentoring pool.

Respondents also expressed dissatisfaction with institutional resources available to support their research activity. Only 27% of participants in our study were satisfied with their program’s research support services. Satisfaction with financial support was generally favourable, though there is room for improvement. These findings align with existing literature, as residents have cited insufficient time, inadequate mentorship, limited faculty support, absence of a structured research curriculum or network, deficiencies in knowledge and skills, lack of incentives and rewards, inadequate research funding, and lack of personal interest in research as barriers to conducting research [23,24,33–39].

Our findings suggest a need for more formal and comprehensive research training during residency. A considerable number of participants believed their lack of research experience prior to residency put them at a disadvantage compared to their peers. Previous reports have highlighted suboptimal teaching of research skills in residency, with adequacy ranging from 19% to 38% [23,33,35]. Recent findings from program directors (PDs) indicate an increase in formal instruction of critical research skills in IM residency programs [29]. While PDs report a marked improvement in research curricula, residents may still perceive these changes as insufficient in preparing them to cope with all aspects of publishing research. This discrepancy likely explains why surveys have consistently reported low rates of resident publications in peer-reviewed journals, ranging from 5% to 10% [23,35]. While more than half of residents expressed confidence in interpreting research findings and interacting effectively with co-authors or reviewers, only 38% felt fully competent in managing all aspects of the publishing process.

Our data suggests there may be specific steps within the publishing process for which residents require further training and support, as only 38% of participants believed they could handle all aspects of publishing. Although, complete research independence may exceed typical residency expectations; programs maybe should aim for fundamental competence with key aspects of the research process. Initial investigations have shown that IM residents have a positive perception of research training that is integrated into their residency experience [40,41]. Incorporating a dedicated research curriculum has been shown to enhance residents’ scholarly output [23,33, 42,43]. Moreover, having a dedicated research director with allocated time has been shown to increase resident publication rates [23,44].

### Well-being

A significant number of residents worked on publications outside of regular work hours resulting in increased stress at home and compromised engagement in leisure activities. Despite previous reports suggesting that work-hour restrictions have allowed more time for academic pursuits and increased research publications, our study highlights the persistent challenge faced by residents in finding sufficient time for research [23,26, 33–36]. Our findings align with recent reports across various specialties [45–47].

Furthermore, sleep was compromised for a substantial number of our participants, highlighting potential negative consequences for well-being. Previous studies have shown that stress and a high risk of burnout, particularly emotional exhaustion, are associated with an increased likelihood of experiencing sleep problems and insomnia [48]. In that regard, one study found that higher institutional research rankings were associated with increased depressive symptoms during intern year; however, the authors acknowledged that this association might reflect other institutional characteristics, such as greater pressure for productivity, institutional culture, or patient complexity [49].

### Quality of research output

Stressors arising from publication pressure can compromise ethical decision-making and lead to scientific misconduct [50–53]. This can include fabrication, falsification, plagiarism, selective reporting, data manipulation, selective citing, and guest authorships [54]. In our study, a considerable proportion of participants (38%) suspected their colleagues had manipulated data due to publication pressure. Similar rates of perceived academic misconduct have been reported in studies involving medical students [55,56]. These behaviours ultimately pose a risk to the integrity of research-based clinical practice [57,58]. Beyond the quality of literature produced, engaging in unprofessional behaviour during training is strongly correlated with a higher likelihood of facing disciplinary action during one’s professional life [59,60].

Our study revealed additional concerning findings related to perceived integrity of the research landscape. Residents perceived that their peers prioritized quantity of research over scientific value. Numerous participants reported publishing solely for the sake of publishing, participating in projects without personal interest, and the perception that publication pressure does not advance medical science. Similar concerns have been documented before, highlighting a trend of prioritizing publishable results at the expense of quality, validity, and scientific rigour [61].

### Clinical training and programmatic requirements

Responses also revealed a self-reported negative impact on clinical training. Many respondents perceived that publication pressure compromised the quality of their clinical work. Cullen et al. found that residents who ultimately matched into cardiology fellowships scored higher on the cardiology section yet lower on the overall PGY-2 ITE, indicating that time devoted to publications and other résumé-building activities for fellowship applications may reduce broad exam preparation [5]. Data suggest that research-focused institutions may also prioritize productivity at the expense of clinical outcomes [49,62,63]. An emphasis on output can have detrimental effects on academic activities that compete with research, including clinical and educational tasks [58,64,65]. Importantly, our findings represent residents’ perceptions rather than objective measures of clinical competence or performance.

Furthermore, over half of our respondents indicated that research expectations influenced their career choices. Similar trends have been observed in other specialties, such as dermatology, where high publication demands have significantly impacted applicants’ success in securing training positions [66–68]. Consequently, specialties with stringent research requirements may unintentionally deter capable trainees who could otherwise excel clinically or educationally.

Participants also recognized some positive effects of research on their training. They reported that research enriched their overall educational experience and observed that colleagues were able to meet their residency requirements despite publication pressure. Similarly, previous studies have shown that residents who undergo research training exhibit enhanced appreciation for evidence-based medicine and achieve better clinical competency scores [69,70].

### How various factors impacted perceptions

The results revealed significant differences in residents’ perceptions based on participant factors, including fellowship match status, race/ethnicity, and future career plans. Residents who had already secured fellowship reported higher levels of research competence and perceived greater positive impact of research on their medical training compared to those who had not yet matched or did not plan to. This observation aligns with a longitudinal study which reported publication rates of 0.29 articles per resident per year for residents bound for fellowships, while non-fellowship-bound residents had a publication rate of 0.13 articles per resident per year [71]. This association may be influenced by increased exposure to research opportunities, greater institutional support, or self-selection of research-oriented residents into competitive fellowship pathways. However, it remains unclear whether residents’ favourable perceptions of research are a result of their success in matching, or the research experience gained during the fellowship application process.

A notable finding was the high research participation rate during residency, despite a significant portion not planning to focus on research in their future careers. Residents who expressed intentions to prioritize research in their future careers rated their research competence, impact of research on well-being, research quality, and impact on their medical training more favourably than those who did not intend to prioritize research after their training. Enhancing awareness of ACGME-compliant scholarly activities, creating pathways towards subspecialty careers that are independent of traditional publication productivity, or alternatively prioritizing the development of research aptitudes ubiquitously for all medical students and residents are steps that could create a more positive experience with research during training.

We observed significant differences in self-perceived research competence based on race, with Black/African American residents reporting higher levels compared to their Asian counterparts. Although our survey design does not allow us to definitively identify the underlying reasons for this finding. Structural inequities in residency training, such as implicit biases, differential expectations, and disparities in mentoring opportunities, have been previously documented and linked to differences in clinical performance assessment scores [72]. Future studies should explore how these structural factors influence residents’ experiences and perceptions of research competence.

### Limitations

This study’s limitations include a cross-sectional design, limiting causal conclusions and temporal insights. Self-reported perceptions introduce potential bias, which we attempted to limit with pilot survey validation. The low overall response rate restricts generalizability. Potential nonresponse bias from PDs forwarding the survey may impact results systematically; those with bias for or against research may have selectively shared the survey. Participants may differ from non-respondents in research experience and perception, introducing selection bias. We acknowledge that some programs may have shared the survey but received no responses; thus, the true response rate could be lower than our calculated figure. This survey, conducted during the COVID-19 pandemic, may reflect the general heightened stress, anxiety, and burnout reported among residents [73,74]. Additionally, even within program categories (e.g. ‘university-based’), institutions vary greatly in resources and research support, highlighting that our classification does not capture the full complexity of training environments. Nevertheless, the 67 participating programs spanned multiple regions and training types, providing diverse perspectives that can yield valuable insights even if they represent only a fraction of the national resident cohort. Exclusive focus on IM residents warrants caution in generalizing findings to other specialties. Moreover, our study specifically assessed residents’ perceptions of publication pressure without direct comparisons to other residency stressors, such as clinical workload or on-call responsibilities, which limits our ability to contextualize the relative impact of publication-related stress.

## Conclusion

This study provides valuable insights into the impact of publication pressure on IM residents. The findings suggest a need to realign expectations for research productivity or target interventions to better support research for all residents, regardless of career goals.

## Supplementary Material

Supplementary Materials.DOC

Survey 5_23_22.pdf

## Data Availability

The data that supports the findings of this study are available from the corresponding author, AK, upon reasonable request.

## References

[1] Acgme. ACGME Program Requirements for Graduate Medical Education in Internal Medicine. Chicago, IL: Accreditation Council for Graduate Medical Education; 2023. Available from: https://www.acgme.org/globalassets/pfassets/programrequirements/140_internalmedicine_2023.pdf.

[2] Results of the 2016 NRMP; 2016 [cited 2023 May 20]; Available from: www.nrmp.org.

[3] McGrail MR, O’Sullivan BG, Bendotti HR, et al. Importance of publishing research varies by doctors’ career stage, specialty and location of work. Postgrad Med J. 2019;95(1122):198–204. Available from: https://academic.oup.com/pmj/article/95/1122/198/6983937 30926718 10.1136/postgradmedj-2019-136473

[4] Solomon SS, Tom SC, Pichert J, et al. Impact of medical student research in the development of physician-scientists. J Investig Med. 2003;51(3):149–156. Available from: https://pubmed.ncbi.nlm.nih.gov/12769197/ doi: 10.1136/jim-51-03-17. 12769197

[5] Cullen MW, Klarich KW, Oxentenko AS, et al. Characteristics of internal medicine residents who successfully match into cardiology fellowships. BMC Med Educ. 2020;20(1):238. Available from: https://pubmed.ncbi.nlm.nih.gov/32723355/ doi: 10.1186/s12909-020-02154-w. 32723355 PMC7385967

[6] Hauer KE, Durning SJ, Kernan WN, et al. Factors associated with medical students’ career choices regarding internal medicine. JAMA [Internet]. 2008;300(10):1154–1164. Available from: https://pubmed.ncbi.nlm.nih.gov/18780844/ doi: 10.1001/jama.300.10.1154. 18780844

[7] Results and Data Specialties Matching Service^®^ 2023 Appointment Year; 2023 [cited 2023 May 20]; Available from: www.nrmp.org.

[8] Oyibo SO. Involving junior doctors in medical article publishing: is it an effective method of teaching? Adv Med Educ Pract. 2017;8:669–674. Available from: https://pubmed.ncbi.nlm.nih.gov/28989295/ doi: 10.2147/AMEP.S147431. 28989295 PMC5624601

[9] Anaya-Prado R, Toledo AH, Toledo-Pereyra LH. The surgeon as a scientific writer. J Invest Surg [Internet]. 2006;19(6):335–339. Available from: https://pubmed.ncbi.nlm.nih.gov/17101601/ doi: 10.1080/08941930601025870. 17101601

[10] Riggs KR, Reitman ZJ, Mielenz TJ, et al. Relationship between time of first publication and subsequent publication success among non-PhD physician-scientists. J Grad Med Educ. 2012;4(2):196–201. Available from: https://pubmed.ncbi.nlm.nih.gov/23730441/ doi: 10.4300/JGME-D-11-00068.1. 23730441 PMC3399612

[11] Schwartz RA, Boyd AS, King J. Medical student publications: a faculty mentor’s perspective. J Am Acad Dermatol. 1997;37(4):667–668. Available from: https://pubmed.ncbi.nlm.nih.gov/9344218/9344218 10.1016/s0190-9622(97)70197-x

[12] Maverakis E, Li CS, Alikhan A, et al. The affect of academic “misrepresentation” on residency match outcomes. Dermatol Online J. 2012;18(1):1. 22301038

[13] FREIDA^TM^ AMA Residency & Fellowship Programs Database [Internet]. [cited 2023 May 20]. Available from: https://freida.ama-assn.org/.

[14] Haven TL, de Goede MEE, Tijdink JK, et al. Personally perceived publication pressure: revising the Publication Pressure Questionnaire (PPQ) by using work stress models. Res Integr Peer Rev. 2019;4(1):7. Available from: https://researchintegrityjournal.biomedcentral.com/articles/10.1186/s41073-019-0066-6 doi: 10.1186/s41073-019-0066-6. 31007948 PMC6454769

[15] Tijdink JK, Smulders YM, Vergouwen ACM, et al. The assessment of publication pressure in medical science; validity and reliability of a Publication Pressure Questionnaire (PPQ). Qual Life Res. 2014;23(7):2055–2062. Available from: https://link.springer.com/article/10.1007/s11136-014-0643-6 doi: 10.1007/s11136-014-0643-6. 24522963

[16] WMA Declaration of Helsinki. Ethical Principles for Medical Research Involving Human Participants – WMA – The World Medical Association [Internet] [cited 2025 Jan 23]. Available from: https://www.wma.net/policies-post/wma-declaration-of-helsinki/. 10.1001/jama.2024.21972 39425955

[17] Nunnally JC, 1924 1982, Bernstein IH. Psychometric theory; 2010 [cited 2023 May 21]; Available from: https://books.google.com/books/about/Psychometric_Theory_3E.html?id=_6R_f3G58JsC.

[18] Rosenberg LE. Young physician-scientists: internal Medicine’s challenge. Ann Intern Med. 2000;133(10):831–832. Available from: https://pubmed.ncbi.nlm.nih.gov/11085847/11085847 10.7326/0003-4819-133-10-200011210-00017

[19] Nih. Physician-Scientist Workforce Working Group Report; 2014. 10.1111/cts.12209 PMC5439807 25123835

[20] Cull WL, Yudkowsky BK, Schonfeld DJ, et al. Research exposure during pediatric residency: influence on career expectations. J Pediatr. 2003;143(5):564–569. Available from: https://pubmed.ncbi.nlm.nih.gov/14615723/ doi: 10.1067/S0022-3476(03)00324-X. 14615723

[21] MacKnin JB, Brown A, Marcus RE. Does research participation make a difference in residency training? Clin Orthop Relat Res. 2014;472(1):370–376. Available from: https://pubmed.ncbi.nlm.nih.gov/23975249/ doi: 10.1007/s11999-013-3233-y. 23975249 PMC3889459

[22] Tooke J, Wass J. Nurturing tomorrow’s clinician scientists. Lancet. 2013;381 (S1):S1–S2. Available from: https://pubmed.ncbi.nlm.nih.gov/23452932/23452932 10.1016/S0140-6736(13)60444-4

[23] Alguire PC, Anderson WA, Albrecht RR, et al. Resident research in internal medicine training programs. Ann Intern Med. 1996;124(3):321–328. Available from: https://pubmed.ncbi.nlm.nih.gov/8554228/ doi: 10.7326/0003-4819-124-3-199602010-00007. 8554228

[24] Hamann KL, Fancher TL, Saint S, et al. Clinical research during internal medicine residency: a practical guide. Am J Med. 2006;119(3):277–283. Available from: https://pubmed.ncbi.nlm.nih.gov/16490480/ doi: 10.1016/j.amjmed.2005.12.001. 16490480

[25] Potti A, Mariani P, Saeed M, et al. Residents as researchers: expectations, requirements, and productivity. Am J Med. 2003;115(6):510–514. Available from: http://www.amjmed.com/article/S0002934303005321/fulltext 14567370 10.1016/j.amjmed.2003.08.017

[26] Namdari S, Baldwin KD, Weinraub B, et al. Changes in the number of resident publications after inception of the 80-hour work week. Clin Orthop Relat Res. 2010;468(8):2278. Available from: /pmc/articles/PMC28952283. 9/ doi: 10.1007/s11999-010-1252-5. 20155407 PMC2895839

[27] Ullrich N, Botelho CA, Hibberd P, et al. Research during pediatric residency: predictors and resident-determined influences. Acad Med. 2003;78(12):1253–1258. Available from: https://pubmed.ncbi.nlm.nih.gov/14660429/ doi: 10.1097/00001888-200312000-00014. 14660429

[28] Bateman EA, Teasell R. Publish or perish. Am J Phys Med Rehabil. 2019;98(12):1142–1146. Available from: https://journals.lww.com/ajpmr/fulltext/2019/12000/publish_or_perish__research_productivity_during.14.aspx doi: 10.1097/PHM.0000000000001299. 31425150

[29] Ercan-Fang NG, Rockey DC, Dine CJ, et al. Resident research experiences in internal medicine residency programs—a nationwide survey. Am J Med. 2017;130(12):1470–1476.e3. Available from: https://pubmed.ncbi.nlm.nih.gov/28919025/ doi:10.1016/j.amjmed.2017.08.033 28919025

[30] Pawar D, Gawde S, Marathe P. Awareness about medical research among resident doctors in a tertiary care hospital: a cross-sectional survey. Perspect Clin Res. 2012;3(2):57. Available from: https://pubmed.ncbi.nlm.nih.gov/22701821/ doi: 10.4103/2229-3485.96446. 22701821 PMC3371549

[31] Adnan NK, Babatunde O, Richard B, et al. Factors influencing resident research: evidence from a 10-year cohort. Int J Clin Biostat Biom. 2020;6(1):1–5. doi: 10.23937/2469-5831/1510025.

[32] Ercan-Fang NG, Mahmoud MA, Cottrell C, et al. Best practices in resident research-a national survey of high functioning internal medicine residency programs in resident research in USA [Internet]. Am J Med Sci. 2021;361(1):23–29. Available from: www.amjmedsci.com doi: 10.1016/j.amjms.2020.08.004. 33288205

[33] Rivera JA, Levine RB, Wright SM. Completing a scholarly project during residency training. Perspectives of residents who have been successful. J Gen Intern Med. 2005;20(4):366–369. Available from: https://pubmed.ncbi.nlm.nih.gov/15857496/ doi: 10.1111/j.1525-1497.2005.04157.x. 15857496 PMC1490090

[34] Gill S, Levin A, Djurdjev O, et al. Obstacles to residents’ conducting research and predictors of publication. Acad Med. 2001;76(5):477. Available from: https://pubmed.ncbi.nlm.nih.gov/11346527/11346527 10.1097/00001888-200105000-00021

[35] Levine RB, Hebert RS, Wright SM. Resident research and scholarly activity in internal medicine residency training programs. J Gen Intern Med. 2005;20(2):155–159. Available from: /pmc/articles/PMC1490049/ doi: 10.1111/j.1525-1497.2005.40270.x. 15836549 PMC1490049

[36] Takahashi O, Ohde S, Jacobs JL, et al. Residents’ experience of scholarly activities is associated with higher satisfaction with residency training. J Gen Intern Med. 2009;24(6):716–720. Available from: https://pubmed.ncbi.nlm.nih.gov/19396500/ doi: 10.1007/s11606-009-0970-4. 19396500 PMC2686770

[37] Chan JY, Narasimhalu K, Goh O, et al. Resident research: why some do and others don’t. Singapore Med J. 2017;58(4):212–217. Available from: /pmc/articles/PMC5392607/ doi: 10.11622/smedj.2016059. 26976220 PMC5392607

[38] Stevenson MD, Smigielski EM, Naifeh MM, et al. Increasing scholarly activity productivity during residency: a systematic review. Acad Med. 2017;92(2):250–266. Available from: https://pubmed.ncbi.nlm.nih.gov/27049539/ doi: 10.1097/ACM.0000000000001169. 27049539

[39] Atreya AR, Stefan M, Friderici JL, et al. Characteristics of Successful Internal Medicine Resident Research Projects: predictors of Journal Publication Versus Abstract Presentation. Acad Med. 2018;93(8):1182–1188. Available from: https://pubmed.ncbi.nlm.nih.gov/29419546/ doi: 10.1097/ACM.0000000000002164. 29419546

[40] Kern DC, Parrino TA, Korst DR. The lasting value of clinical skills. JAMA [Internet]. 1985;254(1):70–76. Available from: https://jamanetwork.com/journals/jama/fullarticle/399316 3999353

[41] Kantor SM, Griner PF. Educational needs in general internal medicine as perceived by prior residents. J Med Educ. 1981;56(9 Pt 1):748–756. Available from: https://pubmed.ncbi.nlm.nih.gov/7277437/ doi: 10.1097/00001888-198109000-00007. 7277437

[42] Schultz HJ. Research during internal medicine residency training: meeting the challenge of the Residency Review Committee. Ann Intern Med. 1996;124(3):340–342. Available from: https://pubmed.ncbi.nlm.nih.gov/8554232/ doi: 10.7326/0003-4819-124-3-199602010-00011. 8554232

[43] Oliver JJ, Ross JM, Davis WT, et al. The development of an emergency medicine resident research program in the united states military. Mil Med. 2019;184(11-12):e622–e625. Available from: https://pubmed.ncbi.nlm.nih.gov/31004142/ doi: 10.1093/milmed/usz071. 31004142

[44] Durning SJ, Cation LJ, Ender PT, et al. A resident research director can improve internal medicine resident research productivity. Teach Learn Med. 2004;16(3):279–283. Available from: https://pubmed.ncbi.nlm.nih.gov/15388386/ doi: 10.1207/s15328015tlm1603_11. 15388386

[45] Mchenry MS, Abramson EL, Mckenna MP, et al. Research in pediatric residency: national experience of pediatric chief residents. Acad Pediatr. 2017;17(2):144–148. doi:10.1016/j.acap.2016.09.010. 28259335

[46] Bammeke F, Liddy C, Hogel M, et al. Family medicine residents’ barriers to conducting scholarly work. Can Fam Physician. 2015;61(9):780–787. Available from: /pmc/articles/PMC4569112/26623463 PMC4569112

[47] Abramson EL, Naifeh MM, Stevenson MD, et al. Research training among pediatric residency programs – a national assessment. Acad Med. 2014;89(12):1674–1680. Available from: /pmc/articles/PMC4315313/ doi: 10.1097/ACM.0000000000000404. 25006705 PMC4315313

[48] Kusurkar RA, van der Burgt SME, Isik U, et al. Burnout and engagement among PhD students in medicine: the BEeP study. Perspect Med Educ. 2021;10(2):110–117. Available from: /pmc/articles/PMC7952475/33284408 10.1007/s40037-020-00637-6 PMC7952475

[49] Pereira-Lima K, Gupta RR, Guille C, et al. Residency program factors associated with depressive symptoms in internal medicine interns: a prospective cohort study. Acad Med. 2019;94(6):869–875. doi: 10.1097/ACM.0000000000002567. 30570500 PMC6538448

[50] Vengoechea J, Moreno S, Ruiz A. Misconduct in medical students. Dev World Bioeth. 2008;8(3):219–225. Available from: https://pubmed.ncbi.nlm.nih.gov/19046259/19046259 10.1111/j.1471-8847.2007.00194.x

[51] Taris TW. Is there a relationship between burnout and objective performance? A critical review of 16 studies. Work Stress [Internet]. 2006;20(4):316–334. Available from: https://www.researchgate.net/publication/233336240_Is_there_a_relationship_between_burnout_and_objective_performance_A_critical_review_of_16_studies doi: 10.1080/02678370601065893.

[52] Dyrbye LN, Massie FS, Eacker A, et al. Relationship between burnout and professional conduct and attitudes among US medical students. JAMA [Internet]. 2010;304(11):1173–1180. Available from: https://pubmed.ncbi.nlm.nih.gov/20841530/ doi: 10.1001/jama.2010.1318. 20841530

[53] Paruzel-Czachura M, Baran L, Spendel Z. Publish or be ethical? Publishing pressure and scientific misconduct in research. Res Ethics. 2021;17(3):375–397. doi: 10.1177/1747016120980562.

[54] Bahl R, Bahl S. Publication pressure versus ethics, in research and publication. Indian J Community Med. 2021;46(4):584–586. doi: 10.4103/ijcm.IJCM_309_20. 35068714 PMC8729298

[55] Dar UF, Khan YS. Self-reported academic misconduct among medical students: perception and prevalence. Sci World J. 2021;2021:5580797. https://www.ncbi.nlm.nih.gov/pmc/articles/PMC8407971/ doi:10.1155/2021/5580797 PMC8407971 34475809

[56] Baldwin DC Jr, Daugherty SR, Rowley BD, Schwarz MD Jr. Cheating in medical school: a survey of second-year students at 31 schools; 1996 undefined. journals.lww.com [Internet]. [cited 2023 Jun 2]. Available from: https://journals.lww.com/academicmedicine/Abstract/1996/03000/Cheating_in_medical_school_a_survey_of.20.aspx. 10.1097/00001888-199603000-00020 8607927

[57] Anderson MS, Ronning EA, De Vries R, et al. The perverse effects of competition on scientists’ work and relationships. Sci Eng Ethics. 2007;13(4):437–461. Available from: https://pubmed.ncbi.nlm.nih.gov/18030595/ doi: 10.1007/s11948-007-9042-5. 18030595

[58] Miller AN, Taylor SG, Bedeian AG. Publish or perish: academic life as management faculty live it. Career Dev Int. 2011;16(5):422–445. Available fromwww.emeraldinsight.com/1362-0436.htm doi: 10.1108/13620431111167751.

[59] Norman NBM, Soo JMP, Lam MYK, et al. Unprofessional behaviour of junior doctors: a retrospective analysis of outcomes by the Singapore Medical Council disciplinary tribunals. Singapore Med J. 2021;62(3):120–125. Available from: https://pubmed.ncbi.nlm.nih.gov/32147740/ doi: 10.11622/smedj.2020021. 32147740 PMC8027145

[60] Foong CC, Holder NAKA, Dutt AR, et al. An intervention to remediate unprofessional behaviours of pre-clinical medical students. EIMJ. 2021;13(2):83–90. doi: 10.21315/eimj2021.13.2.7.

[61] Adler NJ, Harzing AW. When knowledge wins: transcending the sense and nonsense of academic rankings. AMLE. 2009;8(1):72–95. Available from: https://www.researchgate.net/publication/275714282_When_Knowledge_Wins_Transcending_the_Sense_and_Nonsense_of_Academic_Rankings doi: 10.5465/amle.2009.37012181.

[62] Balch CM, Freischlag JA, Shanafelt TD. Stress and burnout among surgeons: understanding and managing the syndrome and avoiding the adverse consequences. Arch Surg. 2009;144(4):371–376. Available from: https://pubmed.ncbi.nlm.nih.gov/19380652/ doi: 10.1001/archsurg.2008.575. 19380652

[63] Shanafelt TD. Enhancing meaning in work: a prescription for preventing physician burnout and promoting patient-centered care. JAMA [Internet]. 2009;302(12):1338–1340. Available from: https://pubmed.ncbi.nlm.nih.gov/19773573/ doi: 10.1001/jama.2009.1385. 19773573

[64] DeAngelis CD. Professors not professing. JAMA [Internet]. 2004;292(9):1060–1061. Available from: https://pubmed.ncbi.nlm.nih.gov/15339898/ doi: 10.1001/jama.292.9.1060. 15339898

[65] van Dalen HP, Henkens K. Intended and unintended consequences of a publish-or-perish culture: a worldwide survey. J Am Soc Inf Sci Technol. 2012;63(7):1282–1293. Available from: https://papers.ssrn.com/abstract=1983205 doi: 10.2139/ssrn.1983205.

[66] Wang JV, Keller M. Pressure to publish for residency applicants in dermatology. Dermatol Online J. 2016;22(3):13030. doi: 10.5070/D3223030382. 27136640

[67] Behbahani S, Dhanda A, Ravikumar V, et al. Dermatology and the Match^®^: An analysis of the number of manuscripts in successful applications. Int J Womens Dermatol. 2020;6(5):431–432. Available from: https://europepmc.org/articles/PMC8060661 doi: 10.1016/j.ijwd.2020.06.008. 33898714 PMC8060661

[68] Stratman EJ, Ness RM. Factors associated with successful matching to dermatology residency programs by reapplicants and other applicants who previously graduated from medical school. Arch Dermatol. 2011;147(2):196–202. Available from: https://pubmed.ncbi.nlm.nih.gov/20956631/ doi: 10.1001/archdermatol.2010.303. 20956631

[69] Smith M. Research in residency: do research curricula impact post-residency practice? Fam Med. 2005;37(5):322–327. Available from: https://europepmc.org/article/med/15883897 15883897

[70] Kohlwes RJ, Shunk RL, Avins A, et al. The PRIME curriculum. Clinical research training during residency. J Gen Intern Med. 2006;21(5):506–509. Available from: https://pubmed.ncbi.nlm.nih.gov/16704399/ doi: 10.1111/j.1525-1497.2006.00438.x. 16704399 PMC1484802

[71] Prasad V, Rho J, Selvaraj S, et al. Publication trends among internal medicine residents and graduates. Am J Med. 2012;125(9):939–944. Available from: https://pubmed.ncbi.nlm.nih.gov/22938929/ doi: 10.1016/j.amjmed.2012.05.014. 22938929

[72] Klein R, Ufere NN, Schaeffer S, et al. Association between resident race and ethnicity and clinical performance assessment scores in graduate medical education. Acad Med. 2022;97(9):1351–1359. Available from: https://pubmed.ncbi.nlm.nih.gov/35583954/ doi: 10.1097/ACM.0000000000004743. 35583954 PMC9910786

[73] Sharma MK, Anand N, Singh P, et al. Researcher burnout: an overlooked aspect in mental health research in times of COVID-19. Asian J Psychiatr. 2020;54:102367. Available from: https://pubmed.ncbi.nlm.nih.gov/33271688/33271688 10.1016/j.ajp.2020.102367 PMC7446612

[74] Gewin V. Pandemic burnout is rampant in academia. Nature. 2021;591(7850):489–491. doi: 10.1038/d41586-021-00663-2. 33723408

