# Supplementary material for: Perceptions of research experience during internal medicine training: insights from a national survey
Source: Ann Med. 2025 Jul 24;57(1):2534848. doi: 10.1080/07853890.2025.2534848 (PMC12291192; doi:10.1080/07853890.2025.2534848)
Supplement: Supplementary Materials.DOC [file IANN_A_2534848_SM8987.doc]

**Supplemental Table S1.** Percentages of Participants Selecting Each Response Category for Perceived Research and Publication Pressure Survey Items, with Corrected Item-Total Correlations and Cronbach's Alpha (n=432).

| Domain | Item | Likert scores | | | | | | | | | CITC | Cronbach’s alpha |
| --- | --- | --- | --- | --- | --- | --- | --- | --- | --- | --- | --- | --- |
|  |  | 1 | 2 | | 3 | | 4 | | | 5 |  |  |
|  |  | SD | | D | | Neither A nor D | | A | SA | |  |  |
| Research Support | My immediate supervisor understands the problems I encounter when I work on my publications | 1.4 | | 17.2 | | 28.7 | | 46.4 | 6.3 | | 0.40 | 0.71 |
|  | I am not satisfied with my program’s research support services (e.g., biostatistics, bioinformatics, IRB, research electives)* | 4.9 | | 21.8 | | 33.1 | | 24.1 | 16.2 | | 0.56 |  |
|  | I am not satisfied with the availability of research projects at my program* | 6.9 | | 25.7 | | 28 | | 24.1 | 15.3 | | 0.59 |  |
|  | When working on a publication, I feel supported by my co-authors | 1.1 | | 11.7 | | 25.2 | | 52.1 | 9.7 | | 0.40 |  |
|  | I am satisfied with the financial support my program offers for research activities (e.g., conference fees, travel stipends, submission fees) | 9.3 | | 17.6 | | 31.8 | | 34.0 | 7.3 | | 0.38 |  |
| Research Competence | I can effectively interpret research data and report my findings | 5 | | 16.4 | | 21.8 | | 49.9 | 11.5 | | 0.52 | 0.74 |
|  | I feel that my lack of research experience prior to residency puts me at a disadvantage compared to my peers* | 6.3 | | 19.7 | | 16.7 | | 34.7 | 22.7 | | 0.46 |  |
|  | I feel confident in the interaction with co-authors, reviewers, and editors | 1.4 | | 13.2 | | 27.2 | | 49.9 | 8.3 | | 0.42 |  |
|  | I can cope with all aspects of publishing my papers | 4.9 | | 30.7 | | 26.4 | | 33.8 | 4.3 | | 0.58 |  |
|  | I am not confident formulating a research question and designing a relevant project* | 6.4 | | 26.9 | | 18.6 | | 39.9 | 8.3 | | 0.57 |  |
| Effect on Medical Training | I suspect that publication pressure leads some colleagues (whether intentionally or not) to cut corners in their clinical work.* | 2.3 | | 13.7 | | 22.7 | | 42.8 | 18.5 | | 0.47 | 0.67 |
|  | My colleagues maintain their residency requirements, despite publication pressure | 2 | | 5.6 | | 21.1 | | 58.8 | 14.4 | | 0.35 |  |
|  | I feel forced to spend time on my publications at the expense of my clinical training* | 6.3 | | 27.5 | | 23.5 | | 33.5 | 9.2 | | 0.50 |  |
|  | I have excluded some career paths because of the amount of research required to be considered competitive* | 6.3 | | 22.5 | | 18.5 | | 27.5 | 25.2 | | 0.40 |  |
|  | Participating in research has enriched my medical training | 4.9 | | 17.2 | | 25.8 | | 43.6 | 8.6 | | 0.41 |  |
| Effect on Well-being | I feel forced to spend time on my publications outside of work hours* | 2.3 | | 12.0 | | 8.6 | | 28.7 | 48.4 | | 0.56 | 0.82 |
|  | I can find sufficient time to work on my publications | 17.2 | | 36.1 | | 24.9 | | 18.9 | 2.9 | | 0.51 |  |
|  | At home, I do not feel stressed about my publications | 26.6 | | 46.1 | | 14.0 | | 10.3 | 2.9 | | 0.61 |  |
|  | I have no peace of mind working on my publications* | 4.6 | | 29.8 | | 26.1 | | 32.1 | 7.4 | | 0.65 |  |
|  | Working on research doesn’t encroach on my ability to enjoy my leisure activities | 27.2 | | 39.8 | | 15.8 | | 15.2 | 2.0 | | 0.60 |  |
|  | My sleep is compromised by my research demands* | 8.0 | | 27.8 | | 20.1 | | 35.0 | 9.1 | | 0.63 |  |
| Research Quality | Publication pressure increases my scientific output, without loss of quality | 10.2 | | 39.8 | | 32.4 | | 16.6 | 9.0 | | 0.42 | 0.64 |
|  | I suspect that for some colleague’s publication pressure leads to data manipulation* | 6.5 | | 23.3 | | 32.2 | | 28.7 | 9.3 | | 0.33 |  |
|  | I believe that publication pressure adds value to medical science | 16.0 | | 38.0 | | 23.8 | | 18.1 | 4.1 | | 0.37 |  |
|  | It is common among residents to publish for the sake of publishing without considering the scientific value of their work* | 0.0 | | 3.7 | | 9.5 | | 44.4 | 42.4 | | 0.52 |  |
|  | I have participated in projects I wasn't interested in for the sake of publication* | 2.9 | | 21.1 | | 9.5 | | 47.8 | 19.7 | | 0.41 |  |

Note. CITC=Corrected item-total Correlations, SA=Strongly Agree, A=Agree, Neither A nor DA= Neither Agree nor Disagree, DA=Disagree, SDA=Strongly Disagree.

* Scores for negatively worded statements

**Supplemental Table S2.** Clinical program residents plan to pursue (n=432).

| **Clinical Field** | **Percent Interested in Field n (%)** |
| --- | --- |
| Adolescent Medicine Fellowship | 1 (0.22 %) |
| Allergy and Immunology Fellowship | 6 (1.29 %) |
| Cardiology Fellowship | 59 (13.73 %) |
| Critical Care Fellowship | 24 (5.58 %) |
| Endocrinology Fellowship | 14 (3.22 %) |
| Gastroenterology Fellowship | 39 (9.01 %) |
| General Medicine/Hospitalist Fellowship | \|  \| \| --- \|  \| 7 (1.72 %) \| \| --- \| |
| Geriatrics Fellowship | 6 (1.50 %) |
| Hematology-Oncology Fellowship | 42 (9.66 %) |
| Hospice and Palliative Care Medicine Fellowship | 5 (1.07 %) |
| Hospitalist Attending | 71 (16.52 %) |
| Infectious Disease Fellowship | 13 (3.00 %) |
| Informatics Fellowship | 2 (0.43 %) |
| Nephrology Fellowship | 13 (3.00 %) |
| Primary Care Attending | 34 (7.94 %) |
| Pulmonary and Critical Care Fellowship | 36 (8.37 %) |
| Pulmonary Fellowship | 10 (2.36 %) |
| Rheumatology Fellowship | 13 (3.00 %) |
| Sports Medicine Fellowship | 5 (1.07 %) |
| Type of Clinical Field Not Listed Here | 6 (1.50 %) |
| Unsure | 24 (5.58 %) |
| Women's Health Fellowship | 1 (0.22 %) |

**Supplemental Table S3.** Those that participated versus those that did not participate in research.

| **Variable** | **Participated n (%)** | **Did Not Participate n (%)** | **p-value** |
| --- | --- | --- | --- |
| **Gender** |  |  |  |
| Female | 178 (51.0 %) | 40 (47.9 %) | 0.388 |
| Male | 167 (47.9 %) | 42 (50.6 %) |  |
| Non-Binary | 2 (0.6 %) | 0 (0.0 %) |  |
| Prefer Not to Say | 2 (0.6 %) | 1 (1.2 %) |  |
| Year of training |  |  |  |
| PGY1 | 88 (25.2 %) | 56 (67.5 %) | <0.001 |
| PGY2 | 139 (39.8 %) | 17 (20.5 %) |  |
| PGY3 | 118 (33.8 %) | 9 (10.8 %) |  |
| PGY4 or greater | 5 (1.2 %) | 0 (0.0 %) |  |
| **Medical School Type** |  |  |  |
| Allopathic US Medical School (MD) | 128 (36.7 %) | 37 (44.6 %) | 0.491 |
| Caribbean Medical School (MD) | 36 (10.3 %) | 9 (10.8 %) |  |
| International Medical School (MD/MBBS) | 122 (35.0 %) | 22 (26.5 %) |  |
| Medical School Type Not Listed Here | 2 (0.6 %) | 0 (0.0 %) |  |
| Osteopathic US Medical School (DO) | 61 (17.5 %) | 15 (18.1 %) |  |
| **Age group** |  |  |  |
| 26-30 years old | 240 (68.9 %) | 57 (68.7 %) | 0.473 |
| 31-35 years old | 93 (26.7%) | 20 (24.1 %) |  |
| 36-40 years old | 8 (2.3 %) | 3 (3.6 %) |  |
| 41-45 years old | 3 (0.9 %) | 0 (0.0 %) |  |
| 46+ years old | 0 (0.0 %) | 1 (1.2 %) |  |
| <25 years old | 5 (1.4 %) | 2 (2.4 %) |  |
| **Race/ethnicity** |  |  |  |
| Asian | 127 (36.4 %) | 31 (37.3 %) | 0.761 |
| Black or African American | 19 (5.4 %) | 4 (4.8 %) |  |
| Hispanic or Latino | 37 (10.6 %) | 6 (7.2 %) |  |
| Native Hawaiian or Other Pacific Islander | 1 (0.3 %) | 1 (1.2 %) |  |
| White | 127 (36.4 %) | 31 (37.3 %) |  |
| **Residency program** |  |  |  |
| Community Based | 85 (24.4 %) | 26 (31.3 %) | 0.293 |
| Community Based, University Affiliated | 90 (25.8 %) | 18 (21.7 %) |  |
| University Based | 174 (49.9 %) | 39 (47.0 %) |  |
| **Has matched into fellowship** | 58 (16.6 %) | 1 (1.2 %) | <0.0001 |
| **In a research track** | 6 (1.7 %) | 1 (1.2 %) | 0.943 |

**Supplemental Table S4.** Means, standard deviations (SD), and one-sample t-test values of scores on each domain of the perceived publication pressure survey (n = 432).

| Domain | Mean | SD | *df* | t |
| --- | --- | --- | --- | --- |
| Research Support | 3.08 | 0.75 | 431 | 2.23 |
| Research Competence | 3.04 | 0.76 | 431 | 0.95 |
| Effect on Medical Training | 2.97 | 0.68 | 431 | -1.01 |
| Effect on Well-Being | 2.45 | 0.79 | 348 | -13.05* |
| Research Quality | 2.42 | 0.64 | 431 | -18.63* |

*p<0.01

**Supplemental Table S5.** Post-hoc comparisons (least significant difference method) to test the differences in internal residents’ perceptions regarding research competence according to their race.

| Race | White | Other | Hispanic or Latino | Black or African American |
| --- | --- | --- | --- | --- |
| Asian | -0.16 | -0.40 | 0.01 | -0.45* |
| Black or African American | 0.30 | 0.05 | 0.46 |  |
| Hispanic or Latino | -0.17 | 0.42 |  |  |
| Other | 0.25 |  |  |  |

* p<0.006

**Supplemental Table S6.** Post-hoc comparisons (least significant difference method) to test the differences in residents’ perceptions regarding the effect of experiencing research on their research competence, well-being, research quality, and effect on medical training according to their plans in making research an important part of their future career.

| Dimension | Future Career | SDA | DA | Neither A nor DA | A |
| --- | --- | --- | --- | --- | --- |
| Research Competence | SA | 0.94* | 0.67* | 0.50* | 0.29 |
|  | A | 0.65* | 0.38* | 0.21 |  |
|  | Neither A nor DA | 0.44* | 0.17 |  |  |
|  | DA | 0.27 |  |  |  |
| Effect on Well-being | SA | 0.71* | 0.35 | 0.08 | 0.14 |
|  | A | 0.56* | 0.21 | -0.07 |  |
|  | Neither A nor DA | 0.63* | 0.28 |  |  |
|  | DA | 0.36 |  |  |  |
| Research Quality | SA | 0.79* | 0.54* | 0.33* | 0.24 |
|  | A | 0.55* | 0.30* | 0.09 |  |
|  | Neither A nor DA | 0.46* | 0.21 |  |  |
|  | DA | 0.25 |  |  |  |
| Effect on Medical Training | SA | 1.07* | 0.70* | 0.39* | 0.35* |
|  | A | 0.72* | 0.35* | 0.04 |  |
|  | Neither A nor DA | 0.70* | 0.30* |  |  |
|  | DA | 0.37* |  |  |  |

Note. SA=Strongly Agree, A=Agree, Neither A nor DA= Neither Agree nor Disagree, DA=Disagree, SDA=Strongly Disagree.

* p<0.006
