# Supplementary material for: Perceptions of research experience during internal medicine training: insights from a national survey
Source: Ann Med. 2025 Jul 24;57(1):2534848. doi: 10.1080/07853890.2025.2534848 (PMC12291192; doi:10.1080/07853890.2025.2534848)
Supplement: Survey 5_23_22.pdf [file IANN_A_2534848_SM8985.pdf]

Resident Experiences With Research

We are conducting the below survey study in order to assess how internal medicine residents experience and attain research. The survey will take approximately 6 minutes to complete. Your participation in this survey is voluntary. All responses will be anonymous. All data collected will be unidentifiable and will be received, stored, and analyzed by Allegheny Health Network in Pittsburgh, PA. At the end of the survey, you will have the option to submit your contact information for a random drawing for one of three $100 Visa gift cards. Your contact information will not be linked in any way to your survey responses. This research has been reviewed and approved by the ASRI-WPAHS Institutional Review Board. If you have any questions or concerns about the project, you may talk to them by calling this toll-free number, 1-844-577-4621.

* Required

1. Which of the following applies to you? *

   ◯ Categorical Internal Medicine Resident

   ◯ Preliminary Internal Medicine Resident

   ◯ Something Else Not Listed Here (Free Text)

2. You selected that your program type was not listed. Please describe it below *

3. What is your current level of training? *

- ◯ PGY1
- ◯ PGY2
- ◯ PGY3
- ◯ PGY4 or greater

4. What is your age group? *

- ◯ <25 years old
- ◯ 26-30 years old
- ◯ 31-35 years old
- ◯ 36-40 years old
- ◯ 41-45 years old
- ◯ 46+ years old
- ◯ Prefer Not To Say

5. To which race/ethnicity do you most identify? *

- ◯ American Indian or Alaska Native
- ◯ Asian
- ◯ Black or African American
- ◯ Hispanic or Latino
- ◯ Native Hawaiian or Other Pacific Islander
- ◯ White
- ◯ Prefer to Self-Describe (Free Text)
- ◯ Prefer Not to Say

6. Please provide your racial/ethnic self-description in the space below *

7. To which gender do you most identify? *

- ◯ Female
- ◯ Male
- ◯ Transgender Female/Trans Woman
- ◯ Transgender Male/Trans Man
- ◯ Non-Binary
- ◯ A Gender Not Listed Here
- ◯ Prefer Not to Say

8. Which best describes your medical school? *

- ◯ Allopathic US Medical School (MD)
- ◯ Caribbean Medical School (MD)
- ◯ International Medical School (MD/MBBS)
- ◯ Osteopathic US Medical School (DO)
- ◯ Medical School Type Not Listed Here

9. Were you required to apply for a visa in order to train in the United States? *

- ◯ Yes
- ◯ No
- ◯ Prefer Not to Say

10. Which description best fits your residency program? *

- ◯ Community Based
- ◯ Community Based, University Affiliated
- ◯ Military Based
- ◯ University Based
- ◯ Something Not Listed Here

11. In which state is your residency program? *

- AL
- AK
- AZ
- AR
- CA
- CO
- CT
- DE
- DC
- FL
- GA
- HI
- ID
- IL
- IN
- IA
- KS
- KY
- LA
- ME
- MD
- MA
- MI
- MN

- ○ MS
- ○ MO
- ○ MT
- ○ NE
- ○ NV
- ○ NH
- ○ NJ
- ○ NM
- ○ NY
- ○ NC
- ○ ND
- ○ OH
- ○ OK
- ○ OR
- ○ PA
- ○ PR
- ○ RI
- ○ SC
- ○ SD
- ○ TN
- ○ TX
- ○ UT
- ○ VT
- ○ VA
- ○ WA

- WV
- WI
- WY

12. Are you currently in a research track e.g. physician scientist training program (PTSP) or research in residency track (RIR)? *

- Yes
- No

13. How many **weeks** of dedicated research time do you anticipate completing by the end of residency? i.e. where research is your primary assignment with little or no clinical responsibilities *

The value must be a number

14. Have you already matched into a fellowship? *

- Yes
- No

15. Which clinical field do you plan to pursue (select all that apply)? *

- ☐ Adolescent Medicine Fellowship
- ☐ Allergy and Immunology Fellowship
- ☐ Cardiology Fellowship
- ☐ Critical Care Fellowship
- ☐ Endocrinology Fellowship
- ☐ Gastroenterology Fellowship
- ☐ General Medicine/Hospitalist Fellowship
- ☐ Geriatrics Fellowship
- ☐ Hematology-Oncology Fellowship
- ☐ Hospice and Palliative Care Medicine Fellowship
- ☐ Hospitalist Attending
- ☐ Infectious Disease Fellowship
- ☐ Informatics Fellowship
- ☐ Nephrology Fellowship
- ☐ Primary Care Attending
- ☐ Pulmonary Fellowship
- ☐ Pulmonary and Critical Care Fellowship
- ☐ Rheumatology Fellowship
- ☐ Sports Medicine Fellowship
- ☐ Women's Health Fellowship
- ☐ Type of Clinical Field Not Listed Here
- ☐ Unsure

16. Which fellowship have you already matched to? *

- ◯ Adolescent Medicine Fellowship
- ◯ Allergy and Immunology Fellowship
- ◯ Cardiology Fellowship
- ◯ Critical Care Fellowship
- ◯ Endocrinology Fellowship
- ◯ Gastroenterology Fellowship
- ◯ General Medicine/Hospitalist Fellowship
- ◯ Geriatrics Fellowship
- ◯ Hematology-Oncology Fellowship
- ◯ Hospice and Palliative Care Medicine Fellowship
- ◯ Infectious Disease Fellowship
- ◯ Informatics Fellowship
- ◯ Nephrology Fellowship
- ◯ Pulmonary Fellowship
- ◯ Pulmonary and Critical Care Fellowship
- ◯ Rheumatology Fellowship
- ◯ Sports Medicine Fellowship
- ◯ Women's Health Fellowship
- ◯ Type of Fellowship Not Listed Here

17. I don’t feel like I could publish more than I already do now
*

- Strongly Agree
- Agree
- Neither Agree nor Disagree
- Disagree
- Strongly Disagree

18. I am not satisfied with my program’s research support (e.g. biostatistics, bioinformatics, IRB, travel stipends, submission fees, research electives etc.)
*

- Strongly Agree
- Agree
- Neither Agree nor Disagree
- Disagree
- Strongly Disagree

19. I am not satisfied with the availability of research projects at my program
*

- Strongly Agree
- Agree
- Neither Agree nor Disagree
- Disagree
- Strongly Disagree

20. I feel that my lack of research experience prior to residency puts me at a disadvantage compared to my peers
*

- Strongly Agree
- Agree
- Neither Agree nor Disagree
- Disagree
- Strongly Disagree

21. I suspect that for some colleagues, publication pressure leads to data manipulation
*

- Strongly Agree
- Agree
- Neither Agree nor Disagree
- Disagree
- Strongly Disagree

22. I believe that publication pressure adds value to medical science
*

- Strongly Agree
- Agree
- Neither Agree nor Disagree
- Disagree
- Strongly Disagree

23. It's common amongst residents to publish for the sake of publishing without considering the scientific value of their work
*

- Strongly Agree
- Agree
- Neither Agree nor Disagree
- Disagree
- Strongly Disagree

24. I suspect that publication pressure leads some colleagues (whether intentionally or not) to cut corners in their clinical work
*

- Strongly Agree
- Agree
- Neither Agree nor Disagree
- Disagree
- Strongly Disagree

25. I plan to make research an important part of my future career
*

- Strongly Agree
- Agree
- Neither Agree nor Disagree
- Disagree
- Strongly Disagree

26. My colleagues maintain their residency requirements, despite publication pressure *

- ◯ Strongly Agree
- ◯ Agree
- ◯ Neither Agree nor Disagree
- ◯ Disagree
- ◯ Strongly Disagree

27. I have excluded some career paths because of the amount of research required to be considered competitive *

- ◯ Strongly Agree
- ◯ Agree
- ◯ Neither Agree nor Disagree
- ◯ Disagree
- ◯ Strongly Disagree

28. I have participated in research during residency (even if not presented or published) *

- ◯ Yes
- ◯ No

29. How many projects have you published **during** residency? *

- 0
- 1-5
- 6-10
- 11-15
- >15

30. I can find sufficient time to work on my publications *

- Strongly Agree
- Agree
- Neither Agree nor Disagree
- Disagree
- Strongly Disagree

31. When working on a publication, I feel supported by my co-authors *

- Strongly Agree
- Agree
- Neither Agree nor Disagree
- Disagree
- Strongly Disagree

32. I can cope with all aspects of publishing my papers
*

- ◯ Strongly Agree
- ◯ Agree
- ◯ Neither Agree nor Disagree
- ◯ Disagree
- ◯ Strongly Disagree

33. My immediate supervisor understands the problems I encounter when I work on my publications
*

- ◯ Strongly Agree
- ◯ Agree
- ◯ Neither Agree nor Disagree
- ◯ Disagree
- ◯ Strongly Disagree

34. I feel confident in the interaction with co-authors, reviewers and editors
*

- ◯ Strongly Agree
- ◯ Agree
- ◯ Neither Agree nor Disagree
- ◯ Disagree
- ◯ Strongly Disagree

35. I feel forced to spend time on my publications outside of work hours
*

- Strongly Agree
- Agree
- Neither Agree nor Disagree
- Disagree
- Strongly Disagree

36. At home, I don't feel stressed about my publications
*

- Strongly Agree
- Agree
- Neither Agree nor Disagree
- Disagree
- Strongly Disagree

37. Working on research doesn't encroach on my ability to enjoy leisure activities
*

- Strongly Agree
- Agree
- Neither Agree nor Disagree
- Disagree
- Strongly Disagree

38. My sleep is compromised by my research demands
*

- Strongly Agree
- Agree
- Neither Agree nor Disagree
- Disagree
- Strongly Disagree

39. I have no peace of mind working on my publications
*

- Strongly Agree
- Agree
- Neither Agree nor Disagree
- Disagree
- Strongly Disagree

40. Publication pressure increases my scientific output, without loss of quality
*

- Strongly Agree
- Agree
- Neither Agree nor Disagree
- Disagree
- Strongly Disagree

41. I have participated in projects I wasn't interested in for the sake of publication
*

- Strongly Agree
- Agree
- Neither Agree nor Disagree
- Disagree
- Strongly Disagree

42. I feel forced to spend time on publications at the expense of my clinical training
*

- Strongly Agree
- Agree
- Neither Agree nor Disagree
- Disagree
- Strongly Disagree

43. Participating in research has enriched my medical training
*

- Strongly Agree
- Agree
- Neither Agree nor Disagree
- Disagree
- Strongly Disagree

44. Please share any additional comments or clarifications about the survey

This content is neither created nor endorsed by Microsoft. The data you submit will be sent to the form owner.

Microsoft Forms
